# Mediterranean-type diet and brain structural change from 73 to 76 years in a Scottish cohort

**DOI:** 10.1212/WNL.0000000000003559

**Published:** 2017-01-31

**Authors:** Michelle Luciano, Janie Corley, Simon R. Cox, Maria C. Valdés Hernández, Leone C.A. Craig, David Alexander Dickie, Sherif Karama, Geraldine M. McNeill, Mark E. Bastin, Joanna M. Wardlaw, Ian J. Deary

**Affiliations:** From the Centre for Cognitive Ageing and Cognitive Epidemiology (M.L., J.C., S.R.C., I.J.D.), Department of Psychology (M.L., J.C., S.R.C., I.J.D.), Brain Research Imaging Centre (M.C.V.H., D.A.D., M.E.B., J.M.W.), Centre for Clinical Brain Sciences (M.C.V.H., D.A.D., M.E.B., J.M.W.), and Scottish Imaging Network, A Platform for Scientific Excellence (SINAPSE) Collaboration, Department of Neuroimaging Sciences (S.R.C., M.C.V.H., D.A.D., M.E.B., J.M.W.), University of Edinburgh; Rowett Institute of Nutrition and Health (L.C.A.C.) and The Institute of Applied Health Sciences (G.M.M.), University of Aberdeen, UK; and Montreal Neurological Institute and Hospital and Douglas Mental Health University Institute (S.K.), McGill University, Canada.

## Abstract

**Objective:**

To assess the association between Mediterranean-type diet (MeDi) and change in brain MRI volumetric measures and mean cortical thickness across a 3-year period in older age (73–76 years).

**Methods:**

We focused on 2 longitudinal brain volumes (total and gray matter; n = 401 and 398, respectively) plus a longitudinal measurement of cortical thickness (n = 323), for which the previous cross-sectional evidence of an association with the MeDi was strongest. Adherence to the MeDi was calculated from data gathered from a food frequency questionnaire at age 70, 3 years prior to the baseline imaging data collection.

**Results:**

In regression models adjusting for relevant demographic and physical health indicators, we found that lower adherence to the MeDi was associated with greater 3-year reduction in total brain volume (explaining 0.5% of variance, *p* < 0.05). This effect was half the size of the largest covariate effect (i.e., age). Cross-sectional associations between MeDi and baseline MRI measures in 562 participants were not significant. Targeted analyses of meat and fish consumption did not replicate previous associations with total brain volume or total gray matter volume.

**Conclusions:**

Lower adherence to the MeDi in an older Scottish cohort is predictive of total brain atrophy over a 3-year interval. Fish and meat consumption does not drive this change, suggesting that other components of the MeDi or, possibly, all of its components in combination are responsible for the association.

Understanding the contributors to healthy aging is an important task, especially if such contributors are modifiable. Diet is modifiable and literature documenting the relationship between a healthy diet and good physical health abounds.^1^ One focus of dietary research has been on the potential merits of a Mediterranean-type diet (MeDi): high consumption of fruit, vegetables, legumes, and cereals, olive oil as the primary source of fat, moderate consumption of fish, low to moderate intake of dairy products and wine (accompanying meals), and low intake of red meat and poultry. Increased adherence to the MeDi has been linked with lower inflammation, better cognitive function, and reduced risk of Parkinson disease and Alzheimer disease and mortality from cardiovascular disease and cancer.^2–6^

Studies of older individuals have shown associations between higher adherence to the MeDi and larger MRI-based brain volumes and cortical thickness.^7–9^ The largest study^7^ found that closer adherence to the MeDi in 468 multiethnic Americans without mild cognitive impairment (mean 80.1 years) was associated with larger (intracranial volume-adjusted) brain volumes. When examining individual food groups, their results indicated that higher fish and lower meat intake were the primary contributors to the observed effects on brain structure. Because their sample was cross-sectional, they could not test for an association with brain loss over time. In the present study, structural brain imaging data were available at 2 timepoints over an interval of roughly 3 years, enabling us to measure the prospective association between the MeDi and meat and fish consumption on multiple indices of brain structural aging.

## METHODS

### Participants.

The Lothian Birth Cohort of 1936 comprises 1,091 participants (49.8% women), all born in 1936, in the Edinburgh region of Scotland, most of whom had completed a mental ability test when they were aged 11 years.^10^ They were aged approximately 70 years at the baseline collection of cognitive and health data. Dietary data were collected at baseline (wave 1) by postal return of questionnaire in 967 participants: 124 were returned blank or not at all. Approximately 3 and 6 years later—waves 2 and 3—the still-available, able, and willing participants underwent a structural MRI brain scan; of those with dietary data, volumetric data were gathered for 562 individuals at wave 2 and 401 at wave 3^11^; see the figure. Age at first MRI scan was 72.65 (±0.72) years and age at second brain scan was 76.36 (±0.64) years; henceforth, these are referred to as 73 and 76, respectively. Individuals lived independently in the community, were relatively healthy, and were free from a diagnosis of dementia at recruitment. Cognitive assessments were made at all 3 timepoints; that is, 2004–2007 (wave 1), 2007–2010 (wave 2), and 2011–2014 (wave 3).

**Figure:**
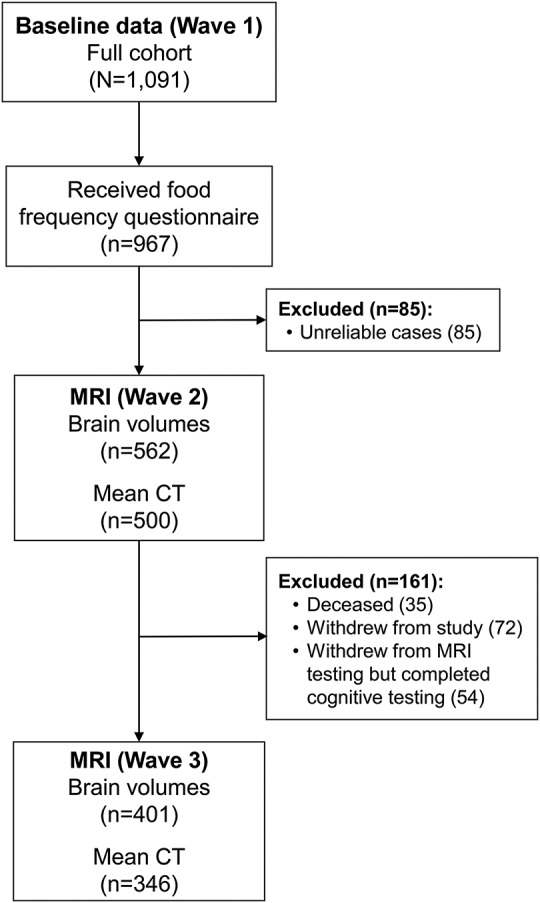
Stages of data collection and sample size of dietary and MRI data in the Lothian Birth Cohort of 1936

### Standard protocol approvals, registrations, and patient consents.

Ethics permission was obtained from the Multi-Centre Research Ethics Committee for Scotland (wave 1: MREC/01/0/56), the Lothian Research Ethics Committee (wave 1: LREC/2003/2/29), and the Scotland A Research Ethics Committee (waves 2, 3: 07/MRE00/58).

### Mediterranean diet.

Data from the Scottish Collaborative Group 168-item Food Frequency Questionnaire, version 7,^12,13^ were used to construct the MeDi score. Exclusions were made for incomplete data (39 had >10 missing items) and for individuals with extreme energy intakes (<2.5th or >97.5th centile, n = 46) to obtain the most reliable food frequency data.^12^ For scoring of the MeDi, we closely followed accepted procedure.^14^ Briefly, individuals were given a value of 1 for each beneficial food component (fruit, vegetables, legumes, cereal, and fish) and a value of 0 for each detrimental component (meat, dairy). The ratio of daily consumption (in grams) of monounsaturated fatty acids to saturated fatty acids was a further beneficial component. Caloric-adjusted sex-specific medians were used as the boundary defining low and high consumption for each of the components. For beneficial components, scores at or above the median were assigned a value of 1, whereas for detrimental components, scores at or above the median were given a value of 0. Moderate alcohol consumption was another positively scored component. It was defined for men as between 10 and 50 g alcohol per day and for women between 5 and 25 g per day. The MeDi score (range 0–9) was calculated by summing the scores for each of the components, with higher scores indicating higher MeDi adherence.

### Covariates.

Education was defined in terms of number of years of full-time education completed. At each test wave, height and weight were clinically measured (to calculate body mass index [BMI]), medical history (stroke, cardiovascular disease, high blood pressure, diabetes) was self-reported at interview, general cognitive ability was indexed by the first unrotated principal component from a battery of cognitive tests,^15^ premorbid IQ was estimated by the National Adult Reading Test (NART),^16^ and current cognitive impairment was assessed by the Mini-Mental State Examination (MMSE).^17^ *APOE* genotyping was performed on genomic DNA isolated from whole blood using TaqMan technology at the Wellcome Trust Clinical Research Facility Genetics Core, Western General Hospital, Edinburgh, UK. *APOE* ε4 allele absence vs presence was used in this study.

### MRI.

The MRI acquisition and processing has been reported previously.^11^ Briefly, a GE (Cleveland, OH) Signa 1.5 T HDXT clinical scanner was used to collect structural T2-, T2*-, fluid-attenuated inversion recovery–, and T1-weighted brain MRI data at both waves. Intracranial volume (ICV) encompassed matter within the inner skull table containing venous sinuses and with a lower limit demarcated as the axial slice just inferior to the inferior boundary of the cerebellar tonsils and on or superior to the superior tip of the odontoid process. A semi-automatic method using T2*-weighted sequence was used to measure ICV with a first estimate gathered automatically using Analyze 9.0 (via the Object Extraction Tool). The cervical spinal cord inferior to the inferior boundary was then manually removed with the pituitary gland (if it had been included). Total brain volume (TBV) represented the difference in volume of the CSF, venous sinuses and meninges, and ICV. Gray matter volume (GMV) was extracted by subtracting CSF, white matter, and white matter hyperintensities from ICV. Variation in head size between individuals was controlled for by using residual scores from the regression of ICV on brain volumes, hereafter referred to as ICV-corrected. We measured cortical thickness using the fully automated CIVET image processing pipeline and qualitative quality control procedures developed at the Montreal Neurologic Institute.^18^ CIVET measures cortical thickness at 81,924 vertices (points where the perpendicular distance between gray matter and white matter surfaces is measured) across the cortex. For clarity, we refer to vertex as the points where cortical thickness is measured, not the cranial vertex. We calculated mean cortical thickness for each participant as the mean of the 81,924 vertex values.

### Statistical analysis.

The main effect of MeDi on brain MRI measures at ages 73 and 76 years was tested using general linear regression where age at MRI testing and sex were controlled for in model 1, and where body mass index (BMI), diabetes, high blood pressure, history of stroke, cardiovascular disease history, years of education, and premorbid IQ were additionally controlled for in model 2. These covariates each showed a significant correlation with at least one of the dependent MRI measures. Education and premorbid IQ (which correlates 0.68 with actual childhood IQ in our sample^19^ but was preferred due to nonmissing data) were considered as potential confounders because they might contribute to differences in diet.^20^ Conversely, current cognitive impairment (Mini-Mental State Examination [MMSE]) and general cognitive ability were measured subsequent to diet, and are partly associated with brain change in this sample^21^; therefore, by adjusting for them, we would remove variation related to brain atrophy. In model 3, we included covariates that aligned best with the largest previously reported analysis of the MeDi and brain MRI measures^7^; these were age at MRI, sex, education, BMI, diabetes, general cognitive ability, and MMSE (to control for mild cognitive impairment). The covariates showed no sign of multicollinearity: the cognitive and education variables showed a maximum correlation of 0.53 with each other, and the variance inflation factor of the models was acceptable. *APOE* ε4 presence, which might relate to variation in brain volume and cortical thickness,^22,23^ was controlled for in supplementary analysis.

For change in MRI measures over time, the same models were repeated using the later MRI residual score (corrected for ICV) as the outcome variable but including the earlier MRI residual score (corrected for age and ICV) as a covariate in all models. This had the effect of removing stable MRI variance across the 2 timepoints, so that the remaining variance in the second MRI outcome measure reflected change and uncorrelated measurement error. The effect of meat and fish consumption on TBV and GMV was further tested to replicate previous findings.^7,9,24^ For the analysis of MRI measures on level scores (ages 73 and 76 years), all covariates collected at that testing wave were used, with the exception of the NART, where the baseline (age 70) measure was considered to be the better estimate of premorbid IQ. For the analysis of MRI change, covariates collected at the later (age 76 years) brain scanning occasion were used, but again with the exception of the NART, where the baseline (age 70) measure was preferred.

## RESULTS

Table 1 shows the demographic, health, and cognitive profile of the full sample and grouped by lower and higher adherence to the MeDi at baseline collection. Whereas we provide significance tests for lower vs higher adherence to the MeDi, such results do not consider confounding from relevant covariates. They are shown primarily for comparison with a previous report^7^; regression analyses are performed using the continuous MeDi scale. The group with greater adherence to the MeDi had more carriers of *APOE* ε4 alleles, and they showed greater TBV and GMV at age 73 years and greater GMV and mean cortical thickness at age 76 years. To test for bias due to sample attrition at wave 3, we compared the mean quantitative MeDi score between those who returned for wave 3 testing (n = 402) vs those who did not (n = 161). No significant difference was found (*t* = −0.42, *df* = 300.81, *p* = 0.67; mean difference of 0.06), nor were differences observed for demographic, cognitive, and health traits, except cognitive ability, where scores were higher (≈0.18 of a SD) for individuals completing both testing waves (*p* = 0.046). See table e-1 at Neurology.org for detailed comparisons.

**Table 1:**
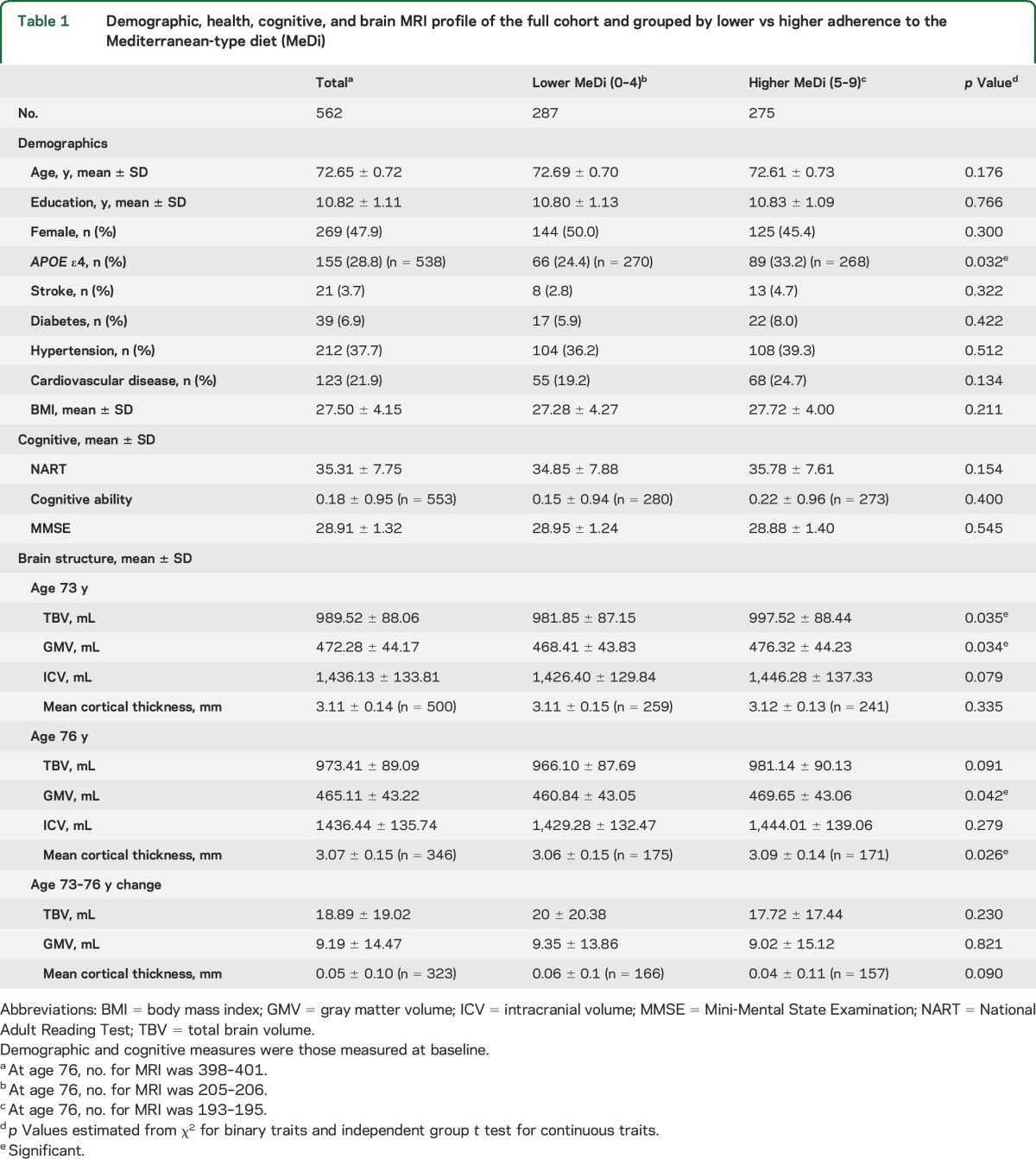
Demographic, health, cognitive, and brain MRI profile of the full cohort and grouped by lower vs higher adherence to the Mediterranean-type diet (MeDi)

In the fully adjusted linear regression models (table 2), the only variable significantly associated with the MeDi was change in TBV (significant in all 3 models). Subsequent analyses of TBV models 2 and 3 adjusting for *APOE* ε4 status produced a larger effect size, although this was based on 16 fewer cases due to missing *APOE* genotyping. The main models 2 and 3 showed respective MeDi standardized effects of 0.068 and 0.058, but, when controlling for *APOE* ε4 presence, effects increased to 0.075 and 0.066, respectively. In model 2, the MeDi effect was the largest after age at MRI and sex and, in model 3, it showed the fifth largest effect. Specific effects of meat and fish consumption on TBV and GMV level (age 73 years) and change (age 76 controlling for age 73) were nonsignificant (table 3), even when accounting for *APOE* ε4 status.

**Table 2:**
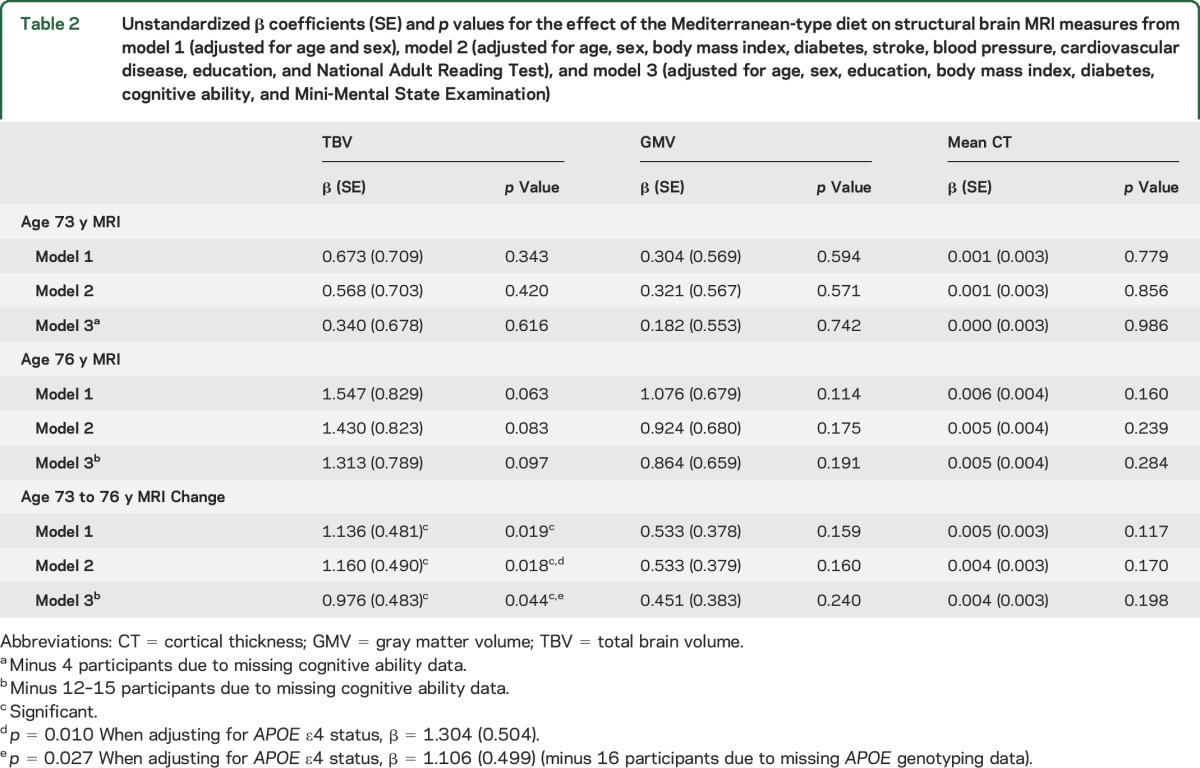
Unstandardized β coefficients (SE) and *p* values for the effect of the Mediterranean-type diet on structural brain MRI measures from model 1 (adjusted for age and sex), model 2 (adjusted for age, sex, body mass index, diabetes, stroke, blood pressure, cardiovascular disease, education, and National Adult Reading Test), and model 3 (adjusted for age, sex, education, body mass index, diabetes, cognitive ability, and Mini-Mental State Examination)

**Table 3:**
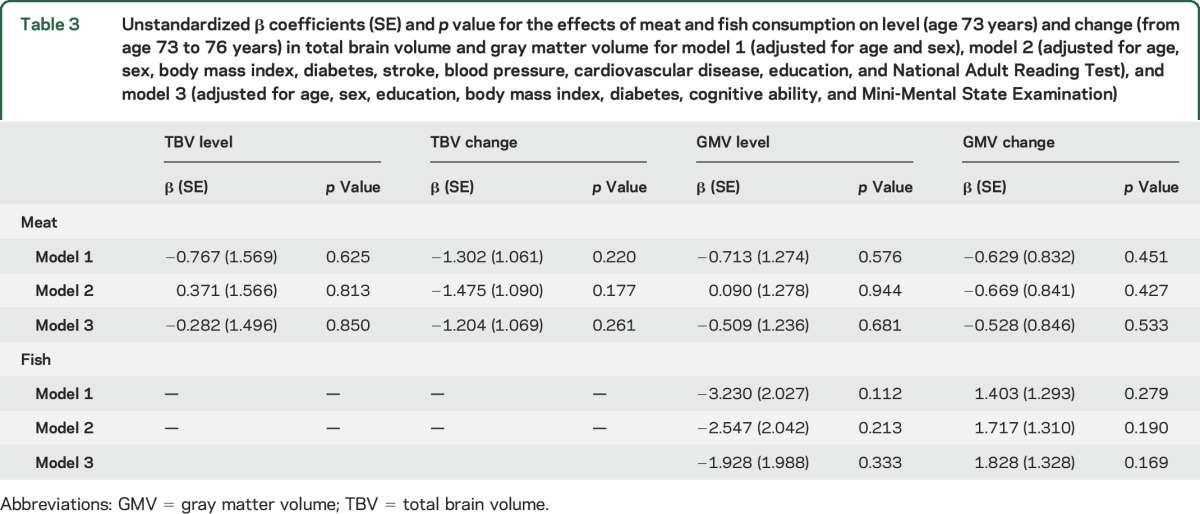
Unstandardized β coefficients (SE) and *p* value for the effects of meat and fish consumption on level (age 73 years) and change (from age 73 to 76 years) in total brain volume and gray matter volume for model 1 (adjusted for age and sex), model 2 (adjusted for age, sex, body mass index, diabetes, stroke, blood pressure, cardiovascular disease, education, and National Adult Reading Test), and model 3 (adjusted for age, sex, education, body mass index, diabetes, cognitive ability, and Mini-Mental State Examination)

## DISCUSSION

Our study investigated MeDi adherence and brain change in older age. We found that greater adherence to the MeDi was associated with lower total brain atrophy over a 3-year period. MeDi was not associated with brain volume or mean cortical thickness measured at ages 73 and 76 years. Fish and meat consumption were not associated with TBV or GMV. Our finding might indicate that greater adherence to the MeDi is protective against total brain atrophy, explaining 0.5% of variance, an effect that was half the size of that due to normal aging in this sample. This effect was not detectable for GMV, suggesting that the effect on TBV was not a consequence of a larger specific influence on gray matter.

Our study also served as a replication of 3 previous studies that measured the effect of the MeDi on MRI measures at a single time point, so it is important to compare their results. Whereas associations between increased adherence to the MeDi and larger TBV, GMV, and WMV were previously reported,^7^ null effects were also found for these same measures in cognitively healthy 75-year-olds from Sweden.^9^ Using voxel-based morphometry, null findings for gray and white matter volumes in 146 older (≥65 years) French participants were also reported.^25^ Our findings are in line with the latter studies, which also focused on ethnically and age homogenous samples. The association between increased adherence to the MeDi and greater cortical thickness reported in a younger (mean age of 54 years) American sample of 52 cognitively healthy individuals^8^ was not replicated in 2 studies (including the present study)^7^ that were almost 10 times larger.

Whereas 2 previous studies^7,9^ found that lower consumption of meat was associated with greater TBV (and also GMV^7^), we do not replicate this effect. Without information on the distribution of meat consumption in these samples, we cannot speculate whether the difference is due to less variation in the quantity of meat consumed or deviation in the type (e.g., poultry vs red; fresh vs processed) of meat consumed in our sample. The previous association between increased fish consumption and larger GMV^7^ was again not replicated here. Differences in the type of fish (oily vs white) consumed between populations might be a factor for the discrepancy in results. With regard to total brain atrophy in our Scottish sample, low meat and high fish intake does not underlie the observed effect of the MeDi. It might be the case that other specific dietary components have direct functional effects on total brain atrophy, but given the interdependency of dietary components, the task of identifying specific foods and potentially nutrients with such an effect is formidable.^26^ Moreover, it has been argued that it is the additive effects of the contributing food components and the ways in which they interact (e.g., through food preparation) that offer increased benefits to health.^27^

In our sample, the *APOE* ε4 allele (Alzheimer disease risk allele) frequency was higher in those who adhered more to the MeDi. None of our sample had been diagnosed with Alzheimer disease so disease progression was unlikely having an influence on diet. In our sensitivity analysis, which additionally adjusted for *APOE* ε4 status, the effect of the MeDi on total brain atrophy became slightly larger. Interestingly, the effect size that we observed for MeDi was larger in the model without adjustment for current cognitive ability and potential dementia, an adjustment that removes part of the variance we are interested in predicting.

The strengths of our study were (1) the sample's age and ethnic homogeneity, which reduced associated confounding with brain change and diet; (2) the collection of MeDi 3 years prior to imaging measures, so that prospective, longer term effects could be evaluated; and (3) the availability of longitudinal imaging data to measure change in brain MRI measures. Potentially limiting is the relatively short time period between brain imaging measurements; a 3-year interval will not tap as much variation in brain change as would much longer intervals, resulting in a smaller detectable effect. Similarly, participants with better general cognitive ability completed repeat MRI testing, which may have constrained variation in associated brain volumes. A correction for multiple testing would deem our result nonsignificant, but the predictor variables were not independent and neither were the brain MRI outcome variables, thus, a correction for number of analyses performed would result in an overly conservative significance criterion. We prefer to focus on the effect size of our novel finding rather than the *p* value; additional studies, and ensuing meta-analyses, are needed to confirm whether the effect we report is reliable.

Our study shows a prospective protective association of the MeDi with brain atrophy in an ethnically and age-homogenous sample of relatively healthy older adults living in Scotland. Diet was measured 3 years before the first brain imaging measurement, so any neurologic disorders correlated with structural brain deficits are unlikely to be influencing diet choice. This might suggest that the MeDi effect is causal. Given that the effect was significant when controlling for education and premorbid IQ also suggests that the MeDi is not simply a function of healthier lifestyle choice in more educated or intelligent individuals, who tend to have larger brain volumes.^28^ A previous study of this cohort found that the association between Mediterranean diet (measured by a latent trait variable from a principal components analysis) and cognitive ability in older age was chiefly due to its relationship with childhood IQ.^20^ Because we sampled diet at a single timepoint, we cannot confirm whether during the period of brain atrophy people were still adhering to the same diet. However, the stability of MeDi scores has been shown to be high over intervals of more than 7 years for those with and without dementia.^6^ If our sample were not still adhering to this diet, that would only highlight the potentially long-lasting protective effects of the MeDi on brain structure during aging. If the stability of diet stretches back much further, then it is possible that diet sets the course for change in brain volume over time, although it does not affect level, which is presumably influenced more by static characteristics of the individual (e.g., genes). That the effect only operates on change is consistent with heritability studies showing that environmental influences (e.g., diet) on total brain volume change (57%) are larger than on level scores of total brain volume (estimates between 3% and 35%).^29,30^ We cannot judge whether longer adherence to the MeDi is associated with greater protection against brain atrophy. Future studies incorporating measures of individuals' lifelong adherence to a particular dietary pattern are needed.

## Supplementary Material

Data Supplement

## GLOSSARY

BMI: body mass index
GMV: gray matter volume
ICV: intracranial volume
MeDi: Mediterranean-type diet
MMSE: Mini-Mental State Examination
NART: National Adult Reading Test
TBV: total brain volume
