# Supplementary material for: Mediterranean-type diet and brain structural change from 73 to 76 years in a Scottish cohort
Source: Neurology. 2017 Jan 31;88(5):449–55. doi: 10.1212/WNL.0000000000003559 (PMC5278943; doi:10.1212/WNL.0000000000003559)
Supplement: Data Supplement [file supp_88_5_449__index.html]

Mediterranean-type diet and brain structural change from 73 to 76 years in a Scottish cohort — Data Supplement 

# Mediterranean-type diet and brain structural change from 73 to 76 years in a Scottish cohort

## Data Supplement

**Neurology® data supplements are not copyedited before publication. Published editorials and translations have been copyedited.  
 © 2017 American Academy of Neurology.  
  
 Files in this Data Supplement:**

- Data Supplement - Microsoft Word file
