# Supplementary material for: Mediterranean-type diet and brain structural change from 73 to 76 years in a Scottish cohort
Source: Neurology. 2017 Jan 31;88(5):449–55. doi: 10.1212/WNL.0000000000003559 (PMC5278943; doi:10.1212/WNL.0000000000003559)
Supplement: Data Supplement [file supp_WNL.0000000000003559_Supplementary_Table_101016.docx]

Supplementary Table e-1. Demographic, Health, and Cognitive Profile of the Group who returned for Wave 2 MRI Testing versus the Group who did not.

|  | Wave 2 Completers | Wave 2 Non-completers | p˜ |
| --- | --- | --- | --- |
| N | 401 | 161 |  |
| **Demographics** |  |  |  |
| Education (mean, SD) | 10.84, 1.12 | 10.77, 1.08 | .507 |
| Female (n, %) | 186, 46.4 | 83, 51.5 | .310 |
| APOE e4 (n, %) | 119, 30.9 (n=385) | 89, 33.2 (n=268) | .110 |
| Stroke | 14, 3.5 | 7, 4.3 | .812 |
| Diabetes | 24, 6 | 15, 9.3 | .222 |
| Hypertension | 155, 38.6 | 57, 35.4 | .534 |
| Cardiovascular disease | 86, 21.4 | 37, 23.0 | .776 |
| BMI | 27.40, 3.87 | 27.73, 4.76 | .434 |
| **Cognitive** |  |  |  |
| NART | 35.54, 7.78 | 34.73, 7.67 | .265 |
| Cognitive Ability | .23, .96 (n=394) | .06, .93 (n=159) | .**046** |
| MMSE | 28.92, 1.40 | 28.91, 1.12 | .923 |
| **MeDi** | 4.42, 1.79 | 4.48, 1.75 | .690 |

Note: Demographic and cognitive measures were those measured at baseline. BMI = body mass index, MMSE = mini mental state examination; ˜ P values estimated from χ² for binary traits and independent group t-test for continuous traits.
